# Healthcare providers’ perceptions of mental and sexual health needs of young males with forced migration experiences in Stockholm Region, Sweden

**DOI:** 10.1186/s12913-025-13991-0

**Published:** 2026-01-10

**Authors:** Fitri Karimah, Jordanos Tewelde McDonald, Maria Stålgren, Mariano Salazar

**Affiliations:** 1 Department of Global Public Health, Karolinska Institutet, Stockholm, Sweden; 2 Transcultural Centre, Stockholm, Sweden; 3 Global and Sexual Health Research Group, Department of Global Public Health, Karolinska Institutet, Stockholm, Sweden

**Keywords:** Healthcare providers, Mental health, Sexual health, Young males with forced migration experiences, Patient-Centered framework

## Abstract

**Background:**

Young males with forced migration experiences (YMFMEs) face numerous health challenges, particularly in terms of sexual and mental health. The disparity in health beliefs and knowledge between healthcare providers (HCPs) and vulnerable groups can exacerbate these challenges. Sexual health encompasses both physical and psychosocial aspects; however, the interplay between these two aspects is often overlooked in research and clinical practice. Additionally, existing research has focused predominantly on females, neglecting the specific needs of YMFMEs. Engaging HCPs provides expert insights into the unique healthcare challenges faced by YMFMEs, enabling the development of tailored, evidence-based interventions that address their specific needs. A patient-centered framework was used to support the interpretation of the data.

**Aim:**

This study aims to explore HCPs’ perceptions of the sexual and mental health needs of YMFMEs and their perspectives on approaches to addressing these needs in the Stockholm Region, Sweden.

**Methods:**

This qualitative study was conducted in the Stockholm Region, Sweden, between May and June 2021 using semi-structured interviews. Nine health care providers were included in the study. The interview transcripts were analyzed using inductive content analysis.

**Results:**

We identified three themes that describe HCPs’ perceptions of YMFMEs’ sexual and mental health needs in healthcare; (1) recognizing and incorporating the biopsychosocial interconnectedness of sexual and mental health into healthcare practices; (2) addressing gaps in health literacy, cultural norms, and expectations is crucial for fostering trust and promoting patient-centered care; and (3) the impact of forced migration and discrimination on YMFMEs’ mental and sexual health and the need for targeted knowledge in healthcare practices.

**Conclusion:**

This study highlights the importance of HCPs understanding the complex relationship between forced migration, mental health, and sexual health for YMFMEs and integrating this awareness into healthcare practices.

**Supplementary Information:**

The online version contains supplementary material available at 10.1186/s12913-025-13991-0.

## Introduction

In 2024, Sweden received 223,204 refugees under the UNHCR mandate, reflecting the country’s ongoing diversification [1]. These individuals have experienced forced migration and fled their countries due to conflict, violence, or fear of persecution, seeking safety in another country [2]. Upon crossing an international border in search of safety, individuals typically must apply for protection from another country, and during this process, they are referred to as asylum seekers [3]. Sweden grants residence permits to asylum seekers recognized as refugees under Swedish legislation and EU regulations [4]. This study explores both groups, asylum seekers and individuals with granted refugee status in Sweden, and we use the term young male with forced migration experiences throughout the study.

Youths, particularly those between the ages of 15 and 24 with asylum-seeking and refugee backgrounds, face extreme and difficult forced migration experiences while transitioning from adolescence to adulthood [5]. Globally, issues related to sexual health and mental health are significant within this group [6, 7]. In general, male youths have difficulty accessing mental health care due to stigma and cultural expectations, such as traditional gender norms, which hinder their ability to be vulnerable and express emotions [8]. In particular, cultural and religious beliefs about mental and sexual concerns may further prevent open discussions among young males with forced migration experiences (YMFMEs) [8–10]. Furthermore, the unique sociocultural challenges they face can limit their access to health resources and information, increasing their lack of understanding and risk of negative health outcomes [9, 11].

The current working definition of sexual health as described by the WHO is “…a state of physical, emotional, mental and social well-being in relation to sexuality; it is not merely the absence of disease, dysfunction or infirmity [12].” Despite the integration of physical, emotional, and social well-being in sexual health as defined by the WHO, considerable youth sexual health research focuses only on physical aspects, such as sexually transmitted diseases (STDs) [13], often treating mental and sexual health as separate domains and overlooking the complex interplay between them [14, 15].

Engaging HCPs to explore their perspectives on the mental and sexual health needs of YMFMEs offers several advantages. First, HCPs bring their expertise and experience to the table, providing valuable insights into the healthcare challenges faced by this demographic [16, 17]. Moreover, by integrating HCPs’ insights, interventions can be tailored to better meet the specific needs and preferences of YMFMEs, promoting more personalized and evidence-based practices [18]. For example, a study in the USA showed that differences in how patients and HCPs describe treatment goals may reflect varying perspectives on the underlying disease processes [18]. While patients prioritize their immediate needs and concerns, HCPs provide expertise in understanding the broader context of diseases and their treatment implications [18].

The sexual and mental health needs of YMFMEs have often been overlooked, as most studies have focused on the needs of young females with forced migration experiences (YFFMEs) [10]. In our previous study, we reported that the sexual and mental health needs of YMFMEs followed a process of individual change over time in Sweden and were influenced by contextual factors such as social and cultural norms and health literacy [19]. In this paper, we aimed to explore HCPs’ perceptions of the sexual and mental health needs of YMFMEs and their perspectives on approaches to addressing these needs in Stockholm Region, Sweden. Understanding how healthcare providers (HCPs) perceive and respond to the sexual and mental health needs of YMFMEs is essential for advancing equitable and effective care for this population. Moreover, our findings can inform the development of training initiatives that enhance HCPs’ capacity to deliver culturally responsive and inclusive services to this vulnerable group.

### Conceptual framework

The patient-centered framework serves as an exemplary model for delivering comprehensive care, especially in addressing the complex health needs of YMFMEs [20]. This service delivery framework emphasizes that quality care must include the following key elements: 1.clear and empathic communication, in which HCPs share and discuss relevant information and care plans with patients; 2. respectful care, ensuring that HCPs are responsive to patients’ preferences, cultural norms, and values; 3. active patient engagement in managing their care through shared decision-making on therapeutic and preventive treatment options, as well as identifying mutual goals for treatment; and 4. effective communication and information sharing among all HCPs involved in the patient’s treatment [20].

This framework is particularly relevant for HCPs working with YMFMEs, as recognizing each patient’s unique sociocultural background and actively involving them in shaping treatment options can enhance adherence and improve treatment outcomes, especially when addressing sensitive issues [21]. To achieve the above, it is essential to explore HCPs’ perceptions of the mental and sexual health needs of this population, as well as their experiences in facilitating care for these issues, and how these challenges can be addressed [21].

## Methods

### Study setting

Between 2013 and 2022, Sweden saw a predominance of refugees from Syria, Afghanistan, Eritrea, Iraq, and Somalia seeking protection, although Ukraine topped the list in 2022 [22]. In Sweden, asylum seekers are entitled to emergency health and dental care, with the option to have an interpreter present if needed. Other free services available under the Swedish Communicable Diseases Act include childbirth, post-partum care, abortion care, and contraception advice [23]. Asylum seekers in Sweden undergo a health assessment upon arrival at the asylum reception, offering advice, tests, and information about healthcare services in Sweden [23, 24]. Those who are granted a residence permit have the same healthcare rights as Swedish citizens [4].

Health services in Stockholm Region, including sexual and reproductive health (SRH) services, are provided mainly through public health care organized by the regions [25]. The Youth Clinic and Men’s Clinic are examples of clinics organized by counties that provide SRH services along with mental health support [25–27]. In addition to the Youth Clinic, there are non-governmental organization (NGO) clinics that focus on mental and sexual health, such as *Riksförbundet*,* för sexuell upplysning;* or the Swedish Association for Sexuality Education (RFSU); and the Origo Clinic, which offers counseling for youth [28, 29]. Despite efforts to improve care, concerns remain regarding entitlement to health care on the basis of migration status and the need for a more comprehensive approach to individual health needs [24, 30].

### Study design and participants’ selection

HCPs were selected based on work experience in delivering mental and sexual health services to YMFMEs. A minimum of 3 years of work experience in a healthcare facility that provides both sexual and mental health services was required. Because of the specific criteria for working in healthcare that provide services related to both concerns, experiences in delivering services to YMFMEs, and the COVID-19 pandemic during the data collection, snowball sampling was used to recruit participants [31]. This meant that the first HCP who met our specific characteristics helped us identify the others.

Our research assistant (MS) and one of the investigators (JTM) initially approached potential participants via email, providing them with detailed information about the study, including its ethical considerations. Those who expressed interest were sent a written informed consent form, which they returned via email prior to data collection. The participants who consented to take part in the study received a store voucher as an appreciation.

The sample size was guided by information power theory, where the size of the sample depended on the aim of the study, sample specificity, use of established theory, quality of dialogue, and analysis strategy [32]. A sample of 6–10 participants with specific characteristics and diverse experiences provided adequate information power [32].

### Data collection

Semi-structured interviews were conducted from May-June 2021. Our research assistant (MS), a Swedish nurse, conducted the interviews in Swedish over Zoom and recorded them for analysis. On average, they lasted one hour. The interview guide explored HCPs’ experiences and perspectives on YMFMEs’ mental and sexual health, focusing on HCPs’ health concerns, needs, knowledge, attitudes, misconceptions, relationships, consent, mental health coping strategies, and experiences with racism were included.

Several questions probed barriers and potential improvements in healthcare services for this population group. The questions were primarily open-ended and exploratory, encouraging detailed responses. The interview guide was developed through an integrative process that combined a comprehensive review of the literature with consultations involving experts in migrant health and clinical practice to ensure contextual relevance and cultural sensitivity. The study included nine HCPs with a range of specialties and years of experience, as summarized in Table 1.

**Table 1:** Demographic characteristics of the participants

**Age**	**Average**
31–60 years old	43.8 years old
**Professions**	
Nurse (including a school nurse)	3
Psychiatric nurse	1
Midwives	3
Psychologist	2
**Years of Experience**	**Average**
3–22 years	8.8 years
**Gender**	
Female	8
Male	1
**Workplace**	
Asylum Reception	3
High School Clinic	1
Youth Clinic	2
Men’s Clinic	1
NGO Clinic	2

### Analysis

The data were analyzed using inductive qualitative content analysis, as described by Graneheim [33]. The first step of the analysis involved multiple thorough readings of the transcripts. JTM reviewed and transcribed the recordings in Swedish, whereas FK translated them into English by using the Word Translate feature. The translated transcripts were reviewed by JTM, a native Swedish speaker.

The coding was initially performed by FK who looked at both the manifest and latent content of the text [33, 34]. The codes were later discussed with the other co-authors (MS and JTM). After the initial coding was performed, codes that had something in common were clustered into categories, which later evolved into themes representing the overall ideas running through the data. The categorization and themes formulation were developed through discussion and consensus agreement. The coding process was conducted in English, utilizing OpenCode 4.03. To illustrate the coding process, Table 2 presents an example of how a participant’s quote was analyzed.

**Table 2:** Example of coding process from interview transcript

Meaning Unit	Codes	Category	Theme
“I also think that these health centers there… uh, that, that you should have a specific, maybe a little specific competence as well. Those who work should have slightly more knowledge and understanding. The patients we meet, what have they been through in the past, where do they come from, and why do they expect this? Perhaps you should be a little sharper, more trained, or have specific experience”.	Lack of specific competence. Need for deeper understanding of patients’ backgrounds. Importance of training. Perceived gap in preparedness.	HCPs’ need for specific knowledge about forced migration’s burden on health.	The impact of forced migration and discrimination on YMFMEs’ mental and sexual health and the need for targeted knowledge in healthcare practices.

We employed several strategies to enhance the trustworthiness of the study [35]. For instance, we provided a thick description of the setting to improve the transferability of our findings. The credibility of our data was strengthened through discussions of the codes, categories, and themes among the authors, who have diverse theoretical and professional backgrounds, thereby enriching the interpretation of the data (researcher triangulation). Additionally, we discussed our results with researchers who were not involved in the study to obtain feedback (peer debriefing).

### Ethical considerations

Ethical approval was obtained from the Swedish Ethical Review Authority, Dnr: 2019 − 01159. The study was conducted in accordance with the Declaration of Helsinki and the Swedish Ethical Review Act. Before participation, our team approached the participants via email and sent them information about the study, including ethical considerations. When the participants agreed to participate, both verbal and written informed consent was obtained. The participants were fully informed about the purpose of the study, emphasizing the voluntary nature of their involvement. To ensure anonymity, pseudonymization of the data was used to protect the participants’ identities. Because the study explores highly sensitive topics such as sexual and mental health, we took additional precautions during transcription and reporting to remove any potentially identifying information and safeguard confidentiality.

## Results

We identified three themes and their corresponding categories describing HCPs’ perceptions of YMFMEs’ sexual and mental health needs related to healthcare (Table 3).

**Table 3:** Themes and categories

Themes	Theme 1: Recognizing and incorporating the biopsychosocial interconnectedness of sexual and mental health into healthcare practices	Theme 2: Addressing gaps in health literacy, cultural norms, and expectations is crucial for fostering trust and promoting patient-centered care	Theme 3: The impact of forced migration and discrimination on YMFMEs’ mental and sexual health and the need for targeted knowledge in healthcare practices.
**Categories**	The link between sexual and mental health identified by HCPs and the need for a holistic approach	Gaps in cultural norms and health literacy create misconceptions and barriers that prevent YMFMEs from seeking care.	Traumatic experiences and discrimination as determinants of the mental and sexual health of YMFMEs
	HCPs’ limited scope in addressing the interconnectedness of sexual and mental health among YMFMEs	Negative experiences and different expectations cause YMFMEs’ lack of trust and underutilization of healthcare	HCPs’ need for specific knowledge about forced migration’s burden on health

### Theme 1: Recognizing and incorporating the biopsychosocial interconnectedness of sexual and mental health into healthcare practices

#### Category 1: The link between sexual and mental health identified by HCPs and the need for a holistic approach

The findings showed that sexual and mental health should not be separated because they often influence each other among YMFMEs. The participants in this study noted that psychological pressure or stress related to the forced migration experience can be a factor in sexual health complaints, or vice versa, where sexual health complaints can lead to mental distress.

*You do an assessment call*,* and you understand that okay*,* this person has erection problems and needs help for that. It is not so strange that they experience sexual illness because there are so many more factors that contribute to*,* uh*,* that there is a general illness*,* a mental illness that you think is the primary thing to address.* (HCP E, Men Clinic).

We noted that the HCPs mentioned that YMFMEs’ sexual health concerns are related to sexual dysfunctions such as erectile dysfunction, premature ejaculation, and low libido. HCPs observed that these concerns are frequently accompanied by internal conflicts surrounding masturbation and premarital sexual activity, areas where mental health support is essential. The participants in this study also mentioned that misconceptions about sexual health can adversely affect the mental well-being of YMFMEs. For example, HCPs observed that this vulnerable group is particularly prone to feelings of guilt, shame, and fear around sexual health, often intensified by strict cultural norms.

This category highlights the importance of recognizing interconnectedness between sexual and mental health in the context of forced migration. HCPs emphasized that YMFMEs’ experiences of psychological distress and sexual health concerns are often intertwined, shaped by complex emotional, cultural, and social factors. Addressing these issues in isolation may overlook underlying causes or reinforce stigma.

#### Category 2: HCPs’ limited scope in addressing the interconnectedness of sexual and mental health among YMFMEs

Although the relationship between sexual and mental health was frequently observed to be interconnected among YMFMEs, this study also revealed several limitations in providing holistic care for them. HCPs working at asylum receptions described that they tended to omit discussions about sexual health with YMFMEs despite discussing it with female asylum seekers. The midwives in this study described their difficulties in discussing mental illness with YMFMEs. The HCPs also often found it difficult to discuss topics such as traumatic experiences, such as sexual assault, during their practice. Although midwives recognized their role in referring patients with overlapping sexual and mental health concerns to psychiatric services, they acknowledged that this process is hindered by the absence of routine mental health screening.

*We do not talk so much about sexual health*,* I will say*,* and that is probably what*,* now when I started to get into this for this interview*,* I started to think about it*,* that we do not talk so much about it*,* more with women then because we talk about contraception and stuff.* (HCP A, asylum reception).

This reflection highlights a gendered gap in care, where sexual health is more readily addressed with females due to topics like contraception, while YMFMEs’ needs remain overlooked. Overall, this category highlights the practical and systemic challenges HCPs face in addressing the interconnected nature of sexual and mental health among YMFMEs. Despite recognizing the link between these domains, providers often encounter barriers such as limited screening routines, discomfort in discussing sensitive topics, and gendered assumptions in care delivery. These constraints may unintentionally result in unequal attention to YMFMEs’ needs, particularly in comparison to female patients.

### Theme 2: Addressing gaps in cultural norms, health literacy, and expectations is crucial for fostering trust and promoting patient-centered care

#### Category 1: Gaps in cultural norms and health literacy create misconceptions and barriers that prevent YMFMEs from seeking care

HCPs recognized and highlighted that cultural norms, conventional understandings of gender roles, and limited access to comprehensive education may influence how YMFMEs perceive and engage with support related to sexual and mental health. One participant commented:

*Beliefs and regulations about sex where you have not been allowed to explore your sexuality on your own terms without it*,* and this may have limited you in your sexual health. What I’m also going to say is the lack of sex education. Ehm*,* you have not had the opportunity to uh*,* assimilate knowledge about the body*,* bodily and sexual functions. In addition*,* they talk about the greater emotional connection to sex and sexuality. Uh*,* so there’s a lot that is*,* that is not verbalized even to oneself or like*,* what to say*,* made conscious to oneself.* (HCP E, Men Clinic).

This quote reflects how constrained access to comprehensive sexual education, coupled with prevailing societal norms, may influence individuals’ capacity to comprehend and articulate their sexual health concerns. Furthermore, our participants revealed the complex beliefs and attitudes of YMFMEs regarding sexual health. The participants in this study reported that YMFMEs’ cultural and religious beliefs often dictate that sexual activities should occur only within the context of marriage, leading to feelings of guilt and inner conflict regarding what is considered right or wrong. According to HCPs’ experience in interacting with YMFMEs, many YMFMEs find it forbidden to discuss sexual health due to religious and cultural restrictions.

*The common thread is low; there is low knowledge about sexuality and relationships. Or*,* a different kind of knowledge*,* and that is what we are not talking about. We often say that they do not have much knowledge about sexuality. They have relationships*,* but they have another. A very religiously influenced*,* traditional*,* conservative*,* very similar to what we had in Sweden a hundred years ago.* (HCP C, psychologist)

HCP C also observed that YMFMEs often approach sexual health issues from philosophical and moral standpoints, making discussions about some areas of some aspects of sexual health easier. HCP C observed that YMFMEs tend to absorb information about STDs more easily, as it is more about anatomy and health education, and provides less room for moralizing, making it the easiest area to educate on. However, discussing sexual health in terms of morals and values becomes challenging, as many YMFMEs believe that sexual activities should occur only within heterosexual marriage and that homosexuality is often seen as a foreign concept.

*In some areas*,* it is easier. STDs*,* anatomy*,* uh*,* and so on. However*,* in some areas*,* it is much more difficult*,* when it comes to morality*,* and when it comes to values.* (HCP C, psychologist)

In addition, deeply ingrained gender norms also make some areas challenging to discuss, particularly consent and contraception. The midwives in this study noted that some YMFMEs may have limited familiarity with the concept of consent in sexual relationships, which can present challenges in clinical discussions. They observed that many YMFMEs come from cultural contexts where traditional gender roles are strongly emphasized, often positioning men as primary decision-makers and women in more subordinate roles. These dynamics may influence how YMFMEs perceive and engage with topics related to sexual autonomy and mutual agreement, highlighting the need for sensitive, respectful dialogue that supports understanding and empowerment.

*So… it is because they often come from patriarchal societies. Uh*,* so*,* so it is like this stereotypical image of relationships. Who’s going to do what*,* and then consent can sometimes be quite difficult to understand*,* because of that.* (HCP G, a midwife)

Our participants argued that contraceptive use is another area that is influenced by traditional gender norms. YMFMEs’ lack of knowledge and misconceptions regarding contraception may be influenced by the tendency to view contraceptive responsibility as primarily belonging to women. In many instances, HCPs observed that YMFMEs were seen to defer decision-making around contraception to their female partners, and some appeared to have a more relaxed stance regarding abortion in the event of an unintended pregnancy.

*Yes*,* it can*,* it can be for example… they said ‘Uh*,* do not have to*,* do not need to wear a condom because… No*,* on the one hand*,* maybe it is the girl’s responsibility* (HCP G, a midwife)

Importantly, we found that traditional gender norms were also noted among HCPs, who often shifted the responsibility of contraception and pregnancy prevention to women. While female asylum seekers and refugees often receive information about contraception, discussions about sexual health with their male counterparts are less common. The lack of discussion in this area is due to HCPs’ omission of sexual health topics during health screening.

*Uh*,* and even though I see myself as a very conscious person*,* I obviously also put that responsibility (contraception) on the woman as well.* (HCP B, asylum reception)

In addition, HCPs reflected on how traditional gender expectations may hinder YMFMEs from openly discussing or seeking support for mental health concerns. Psychologists and psychotherapists involved in this study observed that YMFMEs often face challenges in articulating their emotional struggles, which can make it difficult to initiate conversations around mental well-being. Based on the experiences shared by HCPs, referrals to psychiatric services are sometimes met with discomfort or perceived stigma, as mental health remains a sensitive and, at times, culturally taboo subject. YMFMEs frequently referenced societal ideals that associate masculinity with strength and emotional restraint, which may further complicate their ability to recognize and address psychological distress.

*There are extremely stereotypical gender roles. In addition*,* the man is the one who is supposed to be like*,* what can I say*,* macho*,* uh*,* should deliver eh… Finances and money; he should have a good status in his working life and should not be subjected to violence and oppression. So… It is very difficult for the self-image to*,* to kind of define it (mental health struggles) right from the start.* (HCP G, a midwife)

Limited access to comprehensive education may shape how YMFMEs perceive and pursue support for sexual and mental health concerns. HCPs in this study noted that YMFMEs are often unfamiliar with psychotherapy as a form of care, and may feel hesitant or reluctant to engage in counseling services.

*Therefore*,* they have been really*,* really hesitant about*,* to… Uh*,* medications. I feel that many people are very skeptical about psychotropic drugs. And*,* so*,* and you do not want it to change your brain.* (HCP D, a school nurse)

Study participants thoughtfully suggested that limited access to trustworthy sources of sexual health education in the home countries of YMFMEs may contribute to the development of misunderstandings surrounding sexual health matters.

*Um*,* there’s no one*,* uh*,* who’s talked about being taught or given information about sexual health. Eh*,* many people have not been to school*,* but even those who have been to school have never had information. So what you have heard is based on what other people have said. Um*,* so very limited knowledge about sexual health.* (HCP I, asylum reception).

This quote highlights how limited access to formal sexual health education both within and outside school settings can leave YMFMEs reliant on informal sources of information, such as peer conversations or community beliefs.

The findings in this category reveal how cultural norms, gender expectations, and limited access to formal health education may shape YMFMEs’ understanding of and engagement with sexual and mental health care. These factors can contribute to internal conflict, hesitancy, and stigma, particularly around topics perceived as sensitive or morally charged. While HCPs demonstrated awareness of these challenges, the data also suggest that some providers may unconsciously reinforce gendered assumptions, for example, by prioritizing sexual health discussions with female patients while overlooking similar needs among YMFMEs. This highlights the importance of ongoing critical reflection among healthcare professionals to identify and challenge implicit biases, ensuring that care practices promote shared responsibility and gender equity in sexual and reproductive health.

#### Category 2: Negative experiences and different expectations cause YMFMEs’ lack of trust and underutilization of healthcare

This study revealed a significant challenge as perceived by HCPs from the language barrier, as interpreters are not available at every clinic, and there is uncertainty about the accuracy of interpretation. The participants who worked at asylum reception expressed concerns about the interpreter’s potential biases, which could affect the quality of the information conveyed.

*Actually*,* first of all*,* those of us who are with an interpreter. It is very difficult because you never know what they’re interpreting*,* how much information will get through and how much information will get through. And*,* if it is the right information or not*,* you still try to confirm by always asking the question again*,* have you understood? Can you explain to me again? Uh*,* then it confirms by repeating*,* then I know aha but then it has got the right information.* (HCP I, asylum reception).

In their interviews, HCP D highlighted that negative experiences with healthcare can occur due to HCPs’ lack of understanding of YMFMEs’ experiences.

*And quite a few people have pretty bad experiences of health care because they have met sometimes like that*,* uh*,* maybe at the health center who maybe does not understand your migration experience at all.* (HCP D, a school nurse)

Language barriers and differing expectations around care emerged as significant challenges in building trust between YMFMEs and healthcare providers. Limited access to trained interpreters and uncertainty about interpretation accuracy can complicate communication, especially during sensitive consultations. In addition, when providers are unfamiliar with the migration-related experiences that shape YMFMEs’ perspectives, patients may feel misunderstood or dismissed.

### Theme 3: The impact of forced migration and discrimination on YMFMEs’ mental and sexual health and the need for targeted knowledge in healthcare practices

#### Category 1: Traumatic experiences and discrimination as determinants of the mental and sexual health of YMFMEs

Our study participants described how the traumatic experiences of YMFMEs in their home countries and during their migration journeys significantly shaped their psychological well-being. The participants in this study often mentioned the uncertainty and threats faced during their journey, along with the possibility of becoming slaves or going without food, exacerbating YMFMEs’ mental health concerns, with PTSD, depression, and integration difficulties being major issues upon arrival in their host countries. Uncertainty of residence status and socioeconomic factors were also often mentioned by HCPs in this study, further contributing to YMFMEs’ mental health concerns. In addition to mental health struggles, YMFMEs face difficulties in finding partners because of reputational issues and discrimination.

*Yes… I mean*,* it is…. There is a picture of uh… It is a kind of stereotype. Partners’ parents*,* absolutely. They are bad guys; dangerous; they come from dangerous countries. Uh*,* they have a dangerous religion. Without even meeting the person.* (HCP G, a midwife).

This quote reveals the HCP’s concern about the presence of prejudiced assumptions that may be directed toward YMFMEs and their families, particularly in relation to their cultural or religious background. The findings in this category underscore how trauma, uncertainty, and discrimination intersect to shape YMFMEs’ mental and sexual health outcomes. Experiences of hardship during migration, combined with challenges in the host country can contribute to psychological distress and hinder integration. Moreover, stigmatizing perceptions and reputational biases may affect YMFMEs’ ability to form relationships and access support.

#### Category 2: HCP’ need for specific knowledge about forced migration’s burden on health

HCPs in this study mentioned that they require specific knowledge about the burden of forced migration on health and migration in general to effectively care for YMFMEs. For example, training is needed to understand the needs of different groups with refugee backgrounds and the unique experiences and challenges that YMFMEs face. Another reflection was that knowledge of the above factors can help HCPs recognize their biases and beliefs which might influence their interactions with YMFMEs.

*I also think that these health centers there….uh*,* that*,* that you should have a specific*,* maybe a little specific competence as well. Those who work should have slightly more knowledge and understanding of what… The patients we meet*,* what have they been through in the past*,* where do they come from*,* and why do they expect this? Perhaps you should be a little sharper*,* more trained*,* or have specific experience. At the asylum reception*,* we have that. However*,* at the health centers in general*,* it does not seem like that.* (HCP A, asylum reception)

This category highlights the expressed need among HCPs for more targeted training and knowledge related to the health impacts of forced migration. Providers acknowledged that without a deeper understanding of YMFMEs’ backgrounds—including trauma, displacement, and cultural expectations—they may struggle to offer appropriate and empathetic care.

## Discussion

Our main findings are reflected in the following themes: (1) recognizing and incorporating the biopsychosocial interconnectedness of sexual and mental health into healthcare practices; (2) addressing gaps in health literacy, cultural norms, and expectations is crucial for fostering trust and promoting patient-centered healthcare; and (3) the impact of forced migration and discrimination on YMFMEs’ mental and sexual health and the need for targeted knowledge in healthcare practices.

Although this study draws exclusively from HCPs’ perspectives, a patient-centered framework was intentionally applied during the analysis to ensure that the interpretation remained grounded in the needs and experiences of YMFMEs. The framework was not used to structure the study design or guide data collection, but rather to support a more empathetic and context-sensitive understanding of the data. By interpreting HCPs’ reflections through a patient-centered lens, we aimed to maintain a focus on the health priorities of YMFMEs, particularly in this study where their voices were not directly represented. This approach helped us identify gaps in care, highlight implicit biases, and emphasize the importance of culturally responsive and inclusive healthcare practices.

### Recognizing and incorporating the biopsychosocial interconnectedness of sexual and mental health into healthcare practices

Our research highlights the biopsychosocial interconnectedness of sexual and mental health among YMFMEs in Stockholm, Region, Sweden, and emphasizes the importance of incorporating these aspects into healthcare practices. The HCPs in this study perceived that misconceptions and stigma surrounding sexual health may exacerbate mental health issues among YMFMEs. This includes feelings of guilt, shame, and fear, as well as inner conflicts regarding sexual activities and sexuality. This finding aligns with a study conducted in Sweden that highlighted how cultural and religious beliefs lead to fear and shame around sexual activities, although they did not mention its correlation with mental health [36].

Another important finding is that healthcare providers do not effectively address at the same time the mental and sexual health issues among YMFMEs. The nurses working at the asylum reception in this study may choose not to discuss sexual health concerns with YMFMEs despite consistently doing so with females during asylum seekers’ health assessments upon arrival. A study in Sweden proposed doubt and thought that it is another HCP’s responsibility as a reason for nurses’ hesitancy in discussing sexual health concerns, which may also apply to the nurses in this study [37].

On the other hand, HCPs working at sexual health-focused clinics may struggle with mental health screening and addressing traumatic events, despite their acknowledgment of referring patients with mental health issues as their role. Many healthcare facilities in Sweden already incorporate both mental and sexual health in one clinic, indicating that the interconnectedness of both concerns is well recognized [26–29]. However, in our study, some nurses who work in asylum reception do not discuss sexual health concerns with men, and midwives in youth clinics do not screen for mental health effectively in practice. A study in Sweden emphasized that mental health concerns, especially violence, are difficult topics for midwives and that they do not feel well-equipped to screen for them [38]. The study also mentioned that the existing mental health screening guide was not implemented by midwives, which is similar to the finding of our study [38].

Recognizing and incorporating the interconnectedness of sexual and mental health into healthcare practices aligns with the biopsychosocial perspective principle within the patient-centered framework, which emphasizes understanding health issues within the broader context of patients’ biological, psychological, and social factors [20]. From this perspective, we suggest that mental and sexual health are interconnected aspects of YMFME’s overall well-being. This may involve screening for both mental and sexual health concerns during assessments and providing integrated interventions that address both aspects simultaneously.

It is important to acknowledge that the majority of HCPs who participated in this study were female, and many reported engaging more frequently in discussions about mental and sexual health with female asylum seekers than with their male counterparts. This pattern may reflect underlying gender dynamics in healthcare interactions, wherein female providers feel more at ease or perceive greater receptivity when addressing sensitive topics with female patients. Conversely, it may indicate that YMFMEs are less inclined to initiate or participate in such discussions, potentially due to cultural norms, stigma, or discomfort associated with cross-gender communication. These gendered dynamics merit further investigation, as they may shape both the accessibility and the quality of psychosocial and sexual healthcare received by YMFMEs.

### Addressing gaps in health literacy, cultural norms, and expectations is crucial for fostering trust and promoting patient-centered care

This study also highlights how cultural and religious beliefs, as well as traditional gender norms, may impact YMFMEs’ perceptions and practices related to sexual and mental health. Struggles with feelings of guilt and taboos surrounding sexual health, particularly due to the belief that sexual activities should occur only within heterosexual marriage, were also observed in a study conducted to map sexual health values among Jordanian and Syrian youth [39]. In this study, HCPs highlighted how these beliefs shape YMFMEs’ perceptions of healthcare services and affect their comfort level when discussing sensitive topics.

Additionally, HCPs in our study frequently noted that prevailing traditional gender norms can present challenges when addressing topics such as consent, contraception, and mental health. These norms often position men as primary decision-makers, while assigning the responsibility for pregnancy prevention predominantly to women. This observation is consistent with findings from other studies, which suggest that the concept of consent may be difficult to fully embrace in contexts shaped by patriarchal values [40]. We found that traditional gender norms are particularly concerning because they are observed not only among YMFMEs but also among some HCPs. For example, an HCP working in asylum reception tends to discuss sexual health only with female asylum seekers.

The participants in this study also reported that traditional gender norms harm YMFMEs’ ability to seek and accept support for their mental health issues. Many HCPs in our study perceived that the notion that men should be “macho” and not oppressed can discourage YMFMEs from expressing emotional vulnerability or acknowledging psychological distress. This finding aligns with a study conducted in Australia, which revealed that traditional masculine ideals discourage emotional expression and cause many stigmas around men and vulnerabilities, with people perceiving seeking help as a weakness [41].

The core concept of the patient-centered framework is to encourage collaboration between HCPs and patients [20]. Involving patients in treatment decisions and asking for their opinions about their health concerns may help empower patients from vulnerable populations and make them feel understood [20, 30]. A lack of understanding of how to provide health services can impact low levels of patient satisfaction and non-compliance with recommended treatment, especially among patients with diverse ethnic and racial backgrounds [30].

These findings can also be interpreted through the lens of Sexual Script Theory, which posits that individuals’ culturally informed understandings of sexuality profoundly shape their sexual behaviours and experiences [42]. Healthcare providers must navigate complex cultural scripts, gender norms, and personal belief systems—not to correct them, but to comprehend them deeply enough to deliver care that is respectful, contextually responsive, and empowering.

### The impact of forced migration and discrimination on YMFMEs’ mental and sexual health and the need for targeted knowledge in healthcare practices

This research also highlights the important role of forced migration and integration in the mental and sexual health needs of YMFMEs and emphasizes the need for HCPs to consider these factors in providing better care for this population. All the participants in this study highlighted the need for specialized training to equip them with the competence to address the mental and sexual health concerns of YMFMEs effectively.

In the Stockholm Region, Sweden, only HCPs working in asylum reception and some HCPs in mental health care have the opportunity to receive training specifically to care for asylum seekers and refugees [43]. The training has a good outcome and increases empathy for vulnerable groups [43]. Unfortunately, we found that other HCPs who also meet and provide services for individuals with asylum-seeking backgrounds, for example, those who work at the Youth Clinics, are not as well equipped to care for YMFMEs. Another principle from the patient-centered framework is seeing the patient as a unique person, which means providing care that is tailored based on individuals’ needs and values [20], as well as taking forced migration burden into account, is essential for better care.

In Region Stockholm, such training is already available and offered free of charge by Transkulturellt Centrum. One example is the course *“Transkulturell hälsa och dess betydelse för ditt kliniska arbete”* (Transcultural Health and Its Relevance to Your Clinical Practice), which addresses how forced migration affects physical and mental health and how to navigate challenges in cross-cultural clinical encounters [44]. We recommend that access to training in transcultural health be expanded beyond specialized units.

## Strengths and limitations

The qualitative approach allowed for an in-depth exploration of HCPs’ perspectives and experiences. Additionally, including multiple HCPs from different backgrounds and settings enhanced the credibility and richness of the findings. While this study provides valuable insights into the complex interplay between mental health, sexual health, and forced migration, several limitations should be acknowledged. The study focused specifically on HCPs’ perspectives and experiences, which may not fully represent the perspectives of YMFMEs themselves regarding their sexual and mental health needs.

While the qualitative approach offers an in-depth understanding of unique needs, it also has limitations, such as the potential for subjective interpretation and difficulty in generalizing findings to a larger population. This study only offers HCPs subjective perspectives on their experiences managing and observing YMFMEs. Additionally, there are notable disadvantages to relying solely on HCPs’ perspectives. One concern is the risk of stereotyping, where the diverse needs and individual differences among YMFMEs may be overlooked, perpetuating biases based on cultural or demographic factors. Moreover, there is a limitation in the depth of understanding when exclusively relying on HCP perspectives. HCPs’ views, influenced by personal experiences and professional biases, may not fully capture the backgrounds and diverse needs of YMFMEs. Therefore, while valuable, HCPs’ perspectives should be complemented by direct engagement with YMFMEs to ensure the development of patient-centered interventions.

## Conclusions and recommendations

Our findings indicate that although HCPs recognized the interconnectedness of YMFMEs’ mental and sexual health needs, both barriers and opportunities remain for improving and integrating care across these domains. Policies that promote the implementation of structured, multidisciplinary training programs can equip HCPs with the skills needed to navigate cultural differences, power dynamics, and communication barriers with greater awareness and empathy, thereby enhancing their ability to address the complex health needs of YMFMEs. Such programs should also examine the impact of forced migration and discrimination on health, challenge gendered assumptions about what topics should be discussed, and address other implicit beliefs that may hinder the delivery of effective, person-centered care.

## Supplementary Information

Below is the link to the electronic supplementary material.

Supplementary Material 1

## Data Availability

Relevant data and information are provided in the manuscript and supplementary materials. Complete transcripts and personal data are not publicly available due to these contained information that could compromise research participant privacy. The deidentified dataset may be requested from the corresponding author (mariano.salazar@ki.se) upon reasonable request.

## Abbreviations

HCPs: Healthcare providers
RSFU: Riksförbundet för sexuell upplysning or The Swedish Association for Sexuality Education
SH: Sexual health
STDs: Sexually transmitted diseases
WHO: World Health Organization
YMFMEs: Young males with forced migration experiences
