# Supplementary material for: Healthcare providers’ perceptions of mental and sexual health needs of young males with forced migration experiences in Stockholm Region, Sweden
Source: BMC Health Serv Res. 2026 Jan 10;26:146. doi: 10.1186/s12913-025-13991-0 (PMC12849360; doi:10.1186/s12913-025-13991-0)
Supplement: Supplementary file 1 — Supplementary Material 1 [file 12913_2025_13991_MOESM1_ESM.docx]

Supplementary File 1

Interview Guide

Hi, my name is ____________ I work at KI, and I am currently collaborating on a project about the mental and sexual health of young migrant men. Today, I would like to talk to you about your experience providing care for this population. I would like to remind you that there are no right or wrong answers. We are just interested in your honest experiences and opinions. I would like to record this conversation to be able to give you my full attention while we talk.

Is it OK? Yes No

For us, sexual health requires that people have a positive and respectful approach to sexuality and sexual relationships, as well as the possibility of having pleasurable and safe sexual experiences that are free of coercion, discrimination, and violence.

To be able to offer young migrants adapted healthcare and public health services, with our study, we want to explore the experiences of health care providers in encountering/providing care to young migrants and their perceptions of the group's needs and knowledge in relation to sexual and mental health.

Young men: 15--25 years.

Migrants: maximum 10 years in Sweden, migration background

| **Topics** | **Questions** |
| --- | --- |
| **Experiences meeting young migrant men** | What has been your experience providing care for young migrant men?  Can you describe in what situations you meet this patient group?  What are your perceptions of young migrant men’s health in general?  What are their main **mental health complains**? (depression, anxiety, other) How so?    What are their main **sexual health complains?** (sexually transmitted infections, unpleasant sexual experiences, pain when having sex, difficulties with erections, etc.). How so?  Can you describe how you talk about **sexual health** with this group of patients? Explore: If they do **not** talk about it. How so? How easy or difficult it is? How so?  Can you describe how you talk about **mental health** with this group of patients? Explore: If they do not talk about it. How so? How easy or difficult it is? How so? |
| **Knowledge and attitudes on sexual health** | From your experience, how do you perceive their attitudes and knowledge on **pregnancy prevention** is? How so?  From your experience, how do you perceive their knowledge on **STI prevention** is? How so?  **Explore**: Attitudes toward condom use.  From your experience what are their main knowledge gaps that health care staff should talk about? How so? |
| **Myths on sexual health** | What are the misconceptions about sexual health that you have encountered in this population?  Explore:  - Misconceptions about masturbation? (for example, you will become crazy if you masturbate, etc.)  - Misconceptions about condom use? (diminish pleasure, etc.)  - Misconceptions about use of other contraceptives? (pills-injections can cause infertility).  - Misconceptions about menstrual periods (they are dirty, a woman cannot be touched during these times, etc. )  - Other? |
| **Relationships and consent for sex** | What do migrant men talk about when they discuss having a girlfriend or boyfriend in Sweden? How so?  Some of the young migrant men that we have talked to have told us that is difficult to get and keep a girlfriend and this has been a source of stress for them.  -What do you think about that?  - Is this something that young men has been discussing with you? How so?  - Do you think they should? How so?  - What will you need to be able to discuss this topic with them?  What do you think is this group understanding **on consent for sex** (young women´s capacity to decide with whom, when and where to have sex)?  Is this something that you have discussed with them? How so? |
| **Attitudes/knowledge mental health** | From your experience, how do you perceive their knowledge on good mental health prevention is? How so?  From your experience, what have you seen are their main coping strategies when confronted with stress, depression, anxiety, or other mental health issues? How so? |
| **Experience with racism** | Some of the young migrant men told us that they have felt rejected by some girls and/or their families because they are migrants or because they the girls´ families think migrants have “bad reputation”.  -What do you think about that?  - Is this something that young men has been discussing with you? How so?  - Do you think they should? How so?  - What will you need to be able to discuss this topic with them? |
| **Other causes** | From your experience what are other issues that impair young migrant men sexual health? How so? What can health care do about it?  From your experience what are other issues that impair young migrant men mental health? How so? What can health care do about it?  What can we do to improve young migrant men access to mental and sexual health services? |
| **Other** | Anything else that you would like to add? |
| **Demographics** | Age  Sex  Profession:  Number of years of providing care for young migrant men: |
